# Alzheimer's disease-like perturbations in HIV-mediated neuronal dysfunctions: understanding mechanisms and developing therapeutic strategies

**DOI:** 10.1098/rsob.200286

**Published:** 2020-12-23

**Authors:** Niraj Kumar Jha, Ankur Sharma, Saurabh Kumar Jha, Shreesh Ojha, Dinesh Kumar Chellappan, Gaurav Gupta, Kavindra Kumar Kesari, Shanu Bhardwaj, Shakti D. Shukla, Murtaza M. Tambuwala, Janne Ruokolainen, Kamal Dua, Sandeep Kumar Singh

**Affiliations:** 1 Department of Biotechnology, School of Engineering and Technology (SET), Sharda University, Greater Noida, UP 201310, India; 2 Department of Life Science, School of Basic Science and Research (SBSR), Sharda University, Greater Noida, UP 201310, India; 3 Department of Pharmacology and Therapeutics, College of Medicine and Health Sciences, PO Box 17666, United Arab Emirates University, Al Ain, United Arab Emirates; 4 Department of Life Sciences, School of Pharmacy, International Medical University, Bukit Jalil, Kuala Lumpur 57000, Malaysia; 5 School of Phamacy, Suresh Gyan Vihar University, Jagatpura, Mahal Road, Jaipur, India; 6 Department of Applied Physics, School of Science, Aalto University, Espoo 00076, Finland; 7 Department of Biotechnology, HIMT, Greater Noida, CCS University, UP, India; 8 Priority Research Centre for Healthy Lungs, Hunter Medical Research Institute (HMRI) and School of Biomedical Sciences and Pharmacy, University of Newcastle, Callaghan, NSW 2308, Australia; 9 School of Pharmacy and Pharmaceutical Sciences, Ulster University, Coleraine, County Londonderry, BT52 1SA, UK; 10 Discipline of Pharmacy, Graduate School of Health, University of Technology Sydney, Sydney, New South Wales 2007, Australia; 11 School of Pharmaceutical Sciences, Shoolini University of Biotechnology and Management Sciences, PO Box 9, Solan, Himachal Pradesh 173229, India; 12 Department of Biomedical Research, Centre of Biomedical Research, SGPGI Campus, Lucknow 226014, UP, India; 13 Biological Science, Indian Scientific Education and Technology Foundation, Lucknow 226002, UP, India

**Keywords:** HIV, Alzheimer's disease, HAND, microglia, neuroinflammation, neurotherapeutics

## Abstract

Excessive exposure to toxic substances or chemicals in the environment and various pathogens, including viruses and bacteria, is associated with the onset of numerous brain abnormalities. Among them, pathogens, specifically viruses, elicit persistent inflammation that plays a major role in Alzheimer's disease (AD) as well as dementia. AD is the most common brain disorder that affects thought, speech, memory and ability to execute daily routines. It is also manifested by progressive synaptic impairment and neurodegeneration, which eventually leads to dementia following the accumulation of Aβ and hyperphosphorylated Tau. Numerous factors contribute to the pathogenesis of AD, including neuroinflammation associated with pathogens, and specifically viruses. The human immunodeficiency virus (HIV) is often linked with HIV-associated neurocognitive disorders (HAND) following permeation through the blood–brain barrier (BBB) and induction of persistent neuroinflammation. Further, HIV infections also exhibited the ability to modulate numerous AD-associated factors such as BBB regulators, members of stress-related pathways as well as the amyloid and Tau pathways that lead to the formation of amyloid plaques or neurofibrillary tangles accumulation. Studies regarding the role of HIV in HAND and AD are still in infancy, and potential link or mechanism between both is not yet established. Thus, in the present article, we attempt to discuss various molecular mechanisms that contribute to the basic understanding of the role of HIV-associated neuroinflammation in AD and HAND. Further, using numerous growth factors and drugs, we also present possible therapeutic strategies to curb the neuroinflammatory changes and its associated sequels.

## 1. Introduction

Alzheimer's disease (AD) is the most common neurological complication, which mainly manifests progressive synaptic impairment and neurodegeneration, following excessive formation and accumulation of amyloid-beta (Aβ) [1,2]. Aβ deposits and hyperphosphorylated Tau (pTau), which interfere with the neuronal organization and their function, play a considerable role in AD progression [3]. Aβ-pathology often involves a variety of signals that interrupt the homeostasis of neurons [4]. Currently, there are no definite data that can demonstrate a causative relationship between neuronal damage following human immunodeficiency virus (HIV) infections and the onset of AD. However, available literature indicates that there are some common factors and pathways modulated in HIV^+^ and AD patients, thus suggestive of some similarities in these two pathologies. Among numerous pathways, neuroinflammation is shown closely related to these disorders and is considered a crucial factor in their development and progression. It has been reported that HIV regulatory proteins such as trans-activator of transcription (Tat), envelope glycoprotein (Gp120), viral protein R (Vpr) and negative factor (Nef) can directly influence the central nervous system (CNS) and activate neuroinflammatory pathways followed by neuronal injury and dysfunction. Additionally, abnormal Aβ deposition, a pathological hallmark of AD has been reported in the individuals suffering from HIV infection. Though the abnormalities associated with Aβ burden are more frequent in the AD brain than HIV-infected individuals, it has been predominantly observed in younger HIV-infected individuals [5,6].

Additionally, blood–brain barrier (BBB) dysfunction associated with HIV-1 infection is considered another cause of neuroinflammation in AD. HIV infiltrates macrophages in the CNS by crossing the BBB. The disrupted BBB in HIV patients has been correlated with toxic Aβ aggregation and other abnormalities resulting from a failure to sort out Aβ peptides [7]. The virus-induced fusion of macrophages causes the formation of giant cells and activation of astrocytes which eventually causes injuries to different components of the brain. The most affected areas are the subcortical structures along with the limbic structures and basal ganglia, and the verotoxins, including HIV proteins Gp41, Gp120, Tat, Vpr and Nef, are accountable for such damage. HIV proteins also may cause axonal damage and breakdown of white matter. These injuries cause a decrease in volume of the brain structures such as the caudate nucleus and basal ganglia, resulting in atrophy of the brain volume and decline in cognition [8–10].

HIV also leads to HIV-associated neurocognitive disorders (HAND), since it has a propensity to cross the BBB and cause neuroinflammation [11–14]. HAND exhibits a spectrum of cognitive deficits and typically affects information processing speed, attention, learning and recall memory among other cognitive functions [15]. HAND also has implications for adherence to antiretroviral (ARV) treatment since it affects prospective memory [16]. The exact route in which HIV causes HAND is not yet well known, although HIV replication (potential mechanisms) in the CNS, principally in the basal ganglia and the adjacent subcortical white matter, is where HIV infection is typically observed [17,18].

Among different cell types in the CNS, neurons have the minimal susceptibility to HIV infection; thus the neuronal impairment is reasonably speculated to result from an infection of neighbouring cells like microglia and macrophages, which exert immune functions in the brain. These infected cells result in the production of viral proteins that have the ability to affect the synapse where communication between neurons occurs. Also, the same viral proteins can induce uninfected macrophages, astrocytes and microglial cells that results in the production of neurotoxins and a variety of inflammatory molecules, causing further damage to neurons [19]. Further, the inflammatory molecules and neurotoxins trigger NMDA receptors and may cause additional damage to the neurons following aggregation of calcium (Ca^2+^) in the neurons which activate the formation of excessive free radicals that contribute to oxidative damage. Among other factors, methamphetamine use or abuse and co-infection with hepatitis C virus (HCV) may aggravate damage caused by HIV, involving activation of macrophages and microglial cells [19].

Like amyloid plaques, neurofibrillary tangles (NFTs) consisting of pTau also occur in people suffering from HIV, particularly in aged individuals [20]. The elevated levels of Tau have been reported to occur at earlier ages in individuals suffering from HIV than in healthy individuals. In HIV-infected individuals, tau phosphorylation results from viral proteins and pro-inflammatory cytokines that may impair amyloidosis and precede the development of tau tangles [21]. Higher expression of pTau in HIV individuals is also correlated with ARV treatment [20]. Many comorbid conditions like chronic substance abuse independent of the direct consequences of HIV also lead to HIV transmission, responsiveness and cognitive difficulties [22]. It is apparent that the linkage and causative mechanisms between neuroinflammation, HIV-CNS neuroinfections, HAND and AD are still not completely understood. Therefore, it is important to understand the fundamental molecular linkage among these pathologies, which may help in understanding pathogenesis and developing therapeutics targeting the pathogenesis events along with additional help in diagnosis and prognosis. In the purview of this, herein we summarize various underlying mechanisms which contribute to HIV-associated neuroinflammation in HAND and AD using synoptic tables and schemes. Additionally, numerous possible therapeutic strategies are also presented, which may have the potential to curb these complications and improve quality of life.

## 2. Human cells involved in HIV-associated neuronal damage

HIV-1 interacts with different cell types (table 1) in the CNS, including resident macrophages, neurons and astrocytes that are reported to be involved in neuronal damage [12,26,27]. In the CNS, resident macrophages, neurons and astrocytes are the primary cell targets for HIV infection. In neurodegenerative processes, the roles of macrophages are crucial due to their resistance and sustenance against the cytopathic effects of HIV-1 [19,28–33]. In the CNS, there are four major types of macrophages: choroid-plexus macrophages, meningeal macrophages, perivascular macrophages and microglia [23,34]. Out of these, perivascular macrophages and microglia are believed to play a crucial role in neuronal damage following the release of inflammatory cytokines [23]. Additionally, viral proteins and neurotoxins also take part in the inflammatory processes, provoking apoptosis and differentiation of astrocytes, and impairing normal neurogenesis [12,24,25]. Further, microglial resident cells play a fundamental role in the pathogenesis of HAND, leading to degenerative changes involving numerous mechanisms. The glial cells upon HIV infection release factors and toxins that aggravate neurons and astrocytes [12,35,36]. Astrocytes are neuroectodermal-derived cells, which support the function and metabolism of neurons, ionic homeostasis into the CNS, control of the state of the neuronal synapses by the uptake of neurotransmitters and tissue repair. These are the important components of the BBB and also regulate the immune responses in the brain [37–39]. In addition, astrocytes can facilitate the virus to persist in the CNS, which aids in maintaining low replication of HIV and establishing a latent infection [40]. Furthermore, in HIV-infected cells, viral factors may enhance the release of other chemoattractants that recruit microglia and monocytes, resulting in aggravation of the neuronal damage. Further, cellular factors like interleukin-1β (IL-1β), interferon gamma (IFN-γ) or tumour necrosis factor alpha (TNF-α) have the potential to activate and reactivate viral replication in latently infected cells [19,41–45].

**Table 1.** The role of human cells in HIV-mediated neuronal damage [12,23–25].

neuronal cell	associated effects	types of infection
neuron	enhances P53 expression	restricted
	enhances caspase activation	
	enhances intracellular Ca^2+^ release	
microglia	induces viral replication	productive
	provokes the release of viral proteins including, gp120, Tat and Vpr	
	increases neurotoxins production and also induces the expression of inflammatory mediators, such as PDGF and QUIN	
astrocyte	enhances the production of neurotoxins	restricted
	downregulates the glutamate uptake	
	enhances BBB permeability	
	enhances intracellular release of glutamate and Ca^2+^	
	evokes the migration of monocytes into the brain	
perivascular macrophage	triggers viral replication	productive
	increases neurotoxins production and induces the expression of inflammatory mediators, such as PDGF and QUIN	
	provokes the release of viral proteins including gp120, Tat and Vpr	
oligodendrocyte	enhances cellular apoptosis	restricted
	enhances intracellular Ca^2+^ levels	
	curtails myelin synthesis	

## 3. The direct and indirect mechanisms of HIV induced-neuronal injury

### 3.1. Direct mechanisms

**Figure 1.**
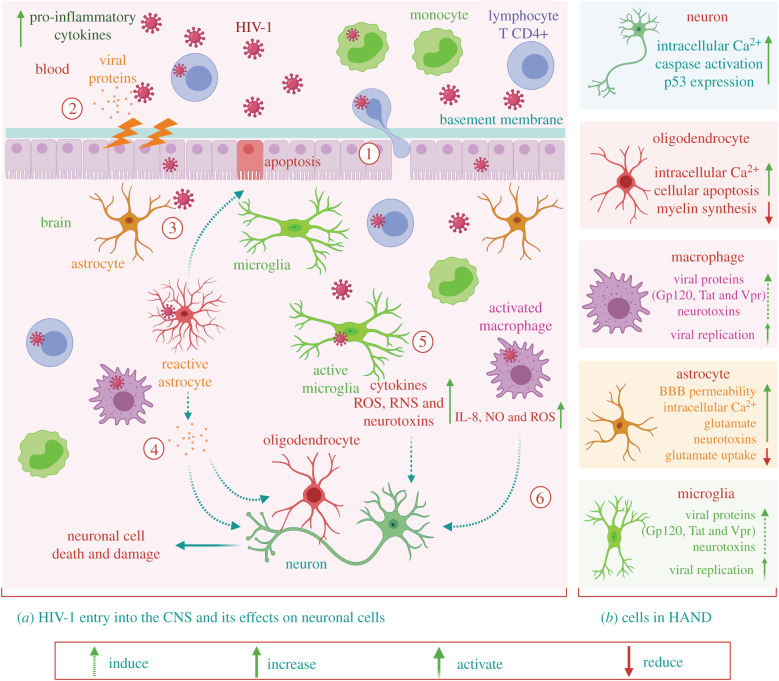
Schematic showing the entry mechanisms of HIV-1 into the CNS and its associated effects on neuronal cells that contribute to neuronal damage and death. (1) HIV-1 can enter through infected T-cells or monocytes that migrate from the bloodstream to the CNS according to the ‘Trojan horse’ hypothesis. (2) The increase in viral proteins and pro-inflammatory cytokines can impair the BBB (epithelial cells) permeability to make virus entry easier. Besides, using infected epithelial cells, virus can reach the other side through a transcytosis process. (3) Reactive astrocytes can provoke epithelial cell apoptosis, leading to the modification of BBB permeability through the release of viral proteins such as Tat. (4) The viral protein Tat has a direct effect on neurons and oligodendrocytes, which cause increased damage and neuronal death. Finally, chronic activation of activated (5) microglia and (6) macrophages causes an increase in the levels of neurotoxins, proinflammatory cytokines, RNA and ROS.

HIV-1 infects CNS involving three different mechanisms (figure 1). In the first mechanism, the virus can directly infect endothelial cells which express the chemokine receptors (CCR3, CXCR4, DC-SIGN) engaged in HIV-1 entry [40,46]. In the second mechanism, the virus may directly cross the impaired BBB due to increased permeability [45,47]. In the third mechanism, according to the ‘Trojan horse’ hypothesis, HIV-1 infected monocytes, perivascular macrophages and leucocytes cross the BBB and release viral particles, which infect resident cells like microglia and lead to persistent infection. This one is believed to be the main mechanism for entry of HIV into the brain, similar to other retroviruses and lentiviruses [40].

Several observations advocate that cells like monocytes are infected before leaving the bone marrow [48]. Particularly, proviral DNA has been observed in these cells with no presence of viral proteins, which facilitated dissemination of the HIV-1 infection [48,49]. An increase in a subset of monocytes, including (CD14^low^CD16^high^), plays a significant role in HIV-1 infection [34,50–54]. These cells display intermediate traits between the differentiated cells (dendritic cells and macrophage) and monocytes [51,53]. Owing to the lower activity of the host restriction factors than the CD14^high^CD16^low^ cells, the cells are more liable to HIV replication following eased permeation through BBB [49–52,54]. Furthermore, viral proteins released into the CNS are believed to induce BBB impairment by enhancing apoptosis and promoting the invasion of HIV as well as other viruses in the different components of the brain [45,55–57].

### 3.2. The indirect mechanisms

In addition to direct mechanisms, HIV-associated neurological complications and neuroinflammation also involve indirect mechanisms such as the infiltration of infected monocytes and lymphocytes in the CNS, release of viral and cellular factors from these infected cells, and infection of the resident cells caused by viral particles released from infected cells or infiltrating into the CNS [58]. The cells (specifically T-cells and monocytes) infected with HIV play a crucial role in the release of pro-inflammatory cytokines that stimulate microglia and astrocytes. The activated microglia and astrocytes along with perivascular macrophages are engaged in releasing inflammatory and neurotoxic mediators, including quinolinic acid (QUIN), nitrogen oxide and platelet-derived growth factor (PDGF), that further lead to neuronal dysfunction and death [45,59].

Despite treatment with ARV agents, a previous study has reported that the level of cytokines such as CCL3, IL-8, CCL2, IFN-γ, CXCL10 and IL-6 was found to be higher in HIV-1 infected individuals in comparison with the uninfected individuals. The higher expressions of cytokines are indicative of uninterrupted neuroinflammation that is accountable for promoting HAND-associated encephalopathy [60]. Recently, Vera et al. [61] reported the presence of neuroinflammatory markers in neuro-asymptomatic HIV-infected patients, despite the effective control of viraemia. The translocation of the virus from the gut to the bloodstream is believed to cause extensive inflammation and altered integrity of white matter, and this reasonably suggests the role of the brain–gut axis in the pathogenesis of HAND [62].

## 4. Detailed mechanisms of neuroinflammation caused by HIV in the brain

HIV is known to play a key role in depleting cluster of differentiation 4 (CD4) cells, and robustly hampering the immune responses. Subsequently, it may rise to opportunistic infections and cause acquired immunodeficiency syndrome (AIDS). HIV is occasionally known as a neurotropic virus, although lacking expression of its main receptor CD4 in neurons; it cannot directly damage the neuronal tissues [63]. Nevertheless, recent phylogenetic analyses showed that HIV could easily access the CNS during primary infection (within the first two weeks), where it can replicate locally and get compartmentalized [64]. Thereafter, virus replication leads to neurotoxicity that is correlated with impaired sensory, cognitive and motor function in patients suffering from HIV, and these neuronal abnormalities are collectively termed HAND [11–14]. These conditions are further categorized into three groups, based on the severity of the symptoms, namely, HIV-associated dementia (HAD), mild neurocognitive disorder (MND) and asymptomatic neurocognitive impairment (ANI) [15]. Patients suffering from these complications exhibit an array of clinical symptoms which may range from cognitive and motor impairment to altered mood and behavioural changes to dementia. The asymptomatic, ANI-HIV^+^ patients have been reported to display greater risk to develop cognitive dysfunctions in comparison with normal patients, and these are considered to reflect the primary stages of AD [65,66]. The incidences of HAND have been found to reduce with the successful establishment of combination antiretroviral therapy (cART) [12,67]. However, despite the availability of cART, the occurrence of HAND is drastically increasing nowadays, generally due to cardiovascular risk factors, increased life expectancy of patients, exposure to environmental hazards and neuroinflammatory changes. Recently, it has been reported that patients diagnosed with HAND with mild/ severe cognitive loss suffer from low quality of life, along with relatively shorter lifespan [68]. Before the introduction of cART, HAD was reported in 15–20% of HIV^+^ patients and was considered a focal risk factor [69,70]. However, following the establishment of this therapy, the total fraction of HAND patients did not show any discrepancy, but the distribution of the classes show alteration with an increase in MND and ANI and a decrease in HAD [12]. Evidence from recent studies shows that neuronal manifestations are becoming more common in the ageing HIV^+^ population [14,71,72]. The data from many clinical trials show poor prediction on the influence of cART on cognitive dysfunction due to BBB restricted lower penetration of the drugs into the CNS. Additionally, some ARV medicines can cause neurotoxicity and are believed to be linked with a poor prediction on the influence of cART on cognitive impairment. Given the available scenario, HAD is also considered as one of the most common forms of dementia in people of less than 40 years of age [14,71,72].

As described previously, HIV uses a mechanism called a ‘Trojan horse’ to enter the CNS, and this mechanism consists of the passage of infected monocytes through the BBB (figure 1) [5,73]. Recently, it has been shown in several clinical studies that CD14^+^CD16^+^ monocytes are competent to easily transmigrate through the BBB, and their high numbers are also reported in HIV-infected patients [5,73]. HIV, once it enters the brain, can damage many cell types, including perivascular macrophages, microglia and potentially adult neural precursors due to the presence of CD4 receptor on these cells [74,75]. Moreover, HIV replication can also be seen in astrocytes in a restrictive manner [76]. Due to these reasons, the brain is sometimes classified as a sanctuary and may serve as a reservoir for HIV [77]. The direct and indirect influences of HIV infection in the brain cause astrocytes and microglia-induced release of cytokines, chemokines and free radicals that result in neuronal dysfunction [12]. In addition, BBB disruption caused by HIV also contributes to further entry/exit of viral proteins and virions.

**Figure 2.**
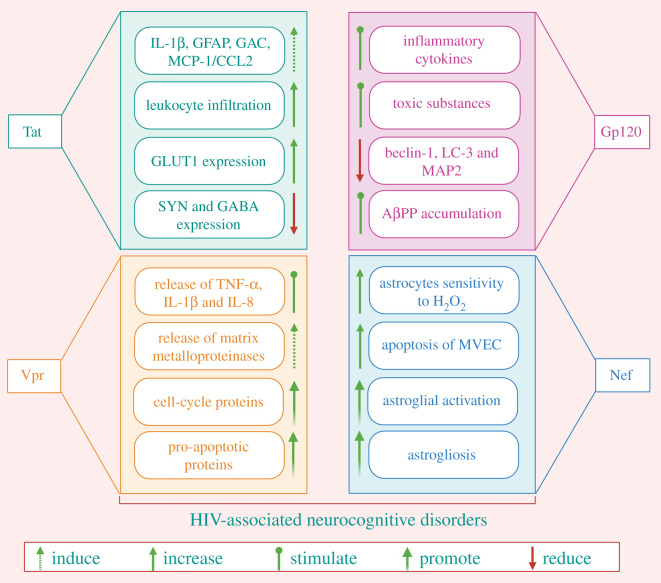
The scheme shows pathological implications of HIV regulatory proteins in neuronal damage. MVEC, microvascular endothelial cells; SYN, synaptophysin; GABA, gamma-aminobutyric acid; GLUT-1, glucose transporter-1.

Numerous HIV regulatory proteins including Tat, Gp120, Vpr and Nef can have direct influences on the nervous system, and these viral proteins are accountable for triggering neuroinflammatory pathways that cause neuronal dysfunction (table 2 and figure 2). The main source of these viral proteins can be infected non-neuronal cells, although these also shed from virions [91,92]. Some viral proteins such as Vpr and Tat are consistently found in the cerebrospinal fluid (CSF) [91,93,94]. Further, the envelope protein Gp120 has been demonstrated to trigger the release of TNF-α and IL-1β, as well as glutamate, which elicits neuronal apoptosis, as evidenced by numerous *ex vivo* and *in vivo* studies [95,96]. Similarly, Tat has been found to potentiate glutamate overactivation of *N*-methyl-d-aspartate receptor (NMDA) receptors and release of cytokines from astrocytes, and potentiate neuronal apoptosis as well [97–99]. Interestingly, Tat and Gp120-induced apoptosis also accounts for higher Ca^2+^ levels when coupled with excitotoxicity events and activated by glutamate deposition in the extracellular spaces. Patients suffering from HIV often have increased levels of glutamate in the CSF, and this correlates well with both the extent of brain atrophy and severity of dementia [100]. Similar to Tat and Gp120, the protein Nef can also trigger cytotoxic effects, though the exact mechanism played by this protein is yet to be investigated [101].

**Table 2.** The roles of HIV regulatory proteins on neuronal damage.

HIV regulatory protein	pathological implications on brain	references
Tat	induces the expression of GAC, GFAP, IL-1β and MCP-1/CCL2	[78–81]
	regulates cellular gene expression	[82]
	enhances the expression of GLUT1 in the hippocampus and cortex; also, enhances leucocyte infiltration	[78,82]
	upregulates the expression of Cx43 human gene	[83]
	decreases SYN expression; also reduces GABA in the cortex.	[82,84]
	interacts with CDK9 and Cyclin T1	[85]
Gp120	activates the release of inflammatory cytokines and toxic substances and accumulation of AβPP	[86,87]
	decreases the expression of MAP2, LC3 and beclin-1	[88]
Vpr	promotes pro-apoptotic and cell-cycle proteins	[57]
	induces the release of matrix metalloproteinases (neurotoxins)	[57]
	provokes the release of IL-1β, TNF-α and IL-8 in macrophages	[57]
Nef	enhances the apoptosis of MVEC; also, enhances the sensitivity of astrocytes to H_2_O_2_	[55,89]
	provokes astrogliosis and astroglial activation	[90]

Furthermore, by regulating microtubule stability, the Vpr induced aggregation of neuronal mitochondria and disrupted axonal transport [102]. In the meantime, it is considered that if the viral load is not checked, there will be a high probability of neuronal dysfunction. Interestingly, HIV-associated neurodegeneration cannot be correlated fully with cognitive deficits, as observed during the early phases of AD [103,104]. In recent studies, cognitive impairments in HAD patients had demonstrated a better correlation with synaptic dysfunction than neurodegeneration, which is further accompanied by synaptic loss, degeneration of axons and astrocytosis [105,106]. More studies are required to demonstrate whether neurocognitive deficits are still observed in patients even when the viral load is well under control. Some cohorts demonstrated that in HIV^+^ viraemic subjects, there is still a high occurrence of HAND, while others suggested that cognition is usually not impaired in individuals with no detectable viraemia [66,107,108]. This can possibly be explained by some possible mechanisms including (i) toxicity of ARVs, (ii) neuroinflammation, (iii) lack of proper cART penetration across the BBB, (iv) increased longevity of infected people and (v) restricted low-noise viral replication [109,110]. Further, constant orchestrated inflammatory events may open up the possibility to understand the linkage between HIV and AD-associated neurodegenerative conditions.

## 5. Mechanisms linking HIV-derived neuronal damage in the AD brain

With the introduction of cART, AIDS has become a chronic disease. A substantial number of HIV^+^ patients over 50–55 years of age are prone to age-related diseases [111]. The plaques formed by extracellular Aβ peptide deposits have been reported in patients, specifically before the cART era. Additionally, accelerated ageing such as immunosenescence is considered an integral part of the natural history of HIV infection. Specifically, HAND makes an impact on an already age-compromised organ and facilitates the occurring rate of neurodegenerative conditions. With reference to AD, concerns have been raised on the potential ties between HIV-CNS infection through various findings highlighting the modulation of amyloid and Tau pathways. Many symptoms correlated with AD pathomechanisms were observed in HIV^+^ individuals. Moreover, similar observations reported in the preclinical models represent neuro-AIDS and mimic neuronal dysfunction in HIV (table 3) [112–133]. It has been reported that CSF features of HIV^+^ patients, present in HAND, resemble the sign and symptoms akin to the early and late stages of AD. For instance, Aβ_1–42_ levels were found considerably altered in the CSF of HAND patients [134]. However, when comparing CSF with HAND, late-stage AD and age-matched controls, reduced Aβ_1–42_ levels were observed in HIV^+^ individuals suffering from neuronal complications [134]. In particular, HIV^+^ patients without neurological manifestations may have a similar range of Aβ_1–42_ levels as reported in non-dementia controls.

**Table 3.** Summary of common neurotoxic mechanisms of AD, observed between experimental models and HIV^+^ patients.

pathological hallmarks/symptoms	observations in *in vivo* and *in vitro* HIV models	methods used	observations in HIV^+^ patients	methods used	references
neurodegeneration	reduction in NeuN	western blotting	loss in cortical grey and white matter of the brain	histologic post-mortem study	[112–114]
	altered neurogenesis	NA	almost 20–50% neuronal damage in the frontal cortex	NA	[115,116]
neuroinflammation	increased expression of microglial Iba1 and astrocytes GFAP	histology and western blotting	peripheral macrophages invasion and chemokines release cause massive gliosis	NA	[112,113,117]
oxidative stress	increased expression of HIF-1, CYP2E1, NAPDH oxydase, IkB and iNOS	western blotting	higher ROS production Impaired mitochondrial dynamics and glucose metabolism	NA	[113,118]
cognitive and learning deficits	deficits in Learning and cognition	morris water maze test	diminished memory performances	NA	[11,119]
atypical Tau phosphorylation	higher expression of p-Ser396, p-Thr181, p-Ser404 and p-Thr231	western blotting	increase in CSF total and phosphorylated Tau	ELISA	[20,112,113,120]
	increased expression of GSK-3β contents and CDK5	western blotting	frontal cortex display increased expression of GSK3b, CDK5 and p35	NA	[112,113,121]
AβPP and Aβ synthesis misprocessing	higher amyloid plaques generation	congo red staining	higher level of CSF Aβ_1–42_	ELISA	[113,120]
	increase of C99 fragment	western blotting	existence of amyloid plaques in brain	NA	[113,122,123]
	higher expression of Aβ_1–42_	eLISA	NA	NA	[124]
activation of neuronal cell death pathways and apoptosis	increase of caspase-3, Bax, pJNK/JNK, Erk contents	western blotting	increased apoptosis	TUNEL assay	[99,113,125,126]
	increased apoptosis	tUNEL assay	increased JNK/ERK contents and activities	kinases assay and western blotting	[99,125]
HPA axis deregulation	higher expression of AVP, CRF mRNA and hypothalamic CRF	NA	impaired cytokine production, modification of glucocorticoid sensitivity and glucocorticoid resistance	NA	[127,128]
	—	—	adrenal insufficiency, elevated plasma GC	NA	[129,130]
blood–brain barrier (BBB)	HIV infection leads to increase leucocytes transmigration through metalloproteinases upregulation and downregulation of TJs proteins	NA	HAD patients show increased CSF/plasma albumin ratio	NA	[76,131]
excitotoxicity	astrocytes cause increase in glutamate release and decrease in glutamate re-uptake	NA	increased levels of CSF glutamate	ELISA	[100,132,133]

Mounting evidence indicates that HIV protein/particle exposure to the brain directly or indirectly influences the regulation of amyloid and Tau signalling pathways [113,135–137]. Recently, neurodegeneration has been noted in murine models of HIV (Gp120 transgenic mice and HIV-1 transgenic rats). It demonstrates increases in oxidative stress, gliosis, apoptosis, abnormal Aβ formation and phosphorylation of Tau. Further, the viral proteins like Tat affect Aβ synthesis, involving numerous mechanisms, including an increase in Aβ synthesis by deregulating structure and function of endolysosomes [135]. Similar to Tat, recombinant Gp120 injected primary hippocampal cells have demonstrated the promotion of Aβ_1–42_ secretion [138]. Also, Tat derived from a lentiviral vector exhibited expression in the hippocampus of transgenic mice (AβPP/PS1) and demonstrated an increase in Aβ_1–42_ formation along with a rise in the volume of amyloid plaques [124]. On the other hand, it causes a rise in Aβ aggregation by inhibiting its mediating degradation enzyme, Neprilysin. Moreover, it also enhances BACE1 expression and synthesis of the C99 fragment to accelerate the production of Aβ [113,135,139]. The increased expressions of BACE1 (commonly observed with AD) have been reported in HIV^+^ patients [140].

Recently, it has been reported that Tat protein in primary hippocampal neuronal cultures forms complexes with toxic Aβ peptides and potentiates a damaging effect by the formation of pores in the membrane [140]. In HIV-1 transgenic rats, the number and volume of amyloid plaques have been reported to be considerably elevated in the cerebral cortex due to an increase in amyloid C-terminal fragment C99 levels (greater than 5-fold) in the brain of HIV-1 transgenic rats [113]. Likewise, HIV-1 infected cells released p17 (HIV-1 matrix protein) which showed participation in Aβ-induced neuronal toxicity ascribed to misfolding and aggregation even when protease inhibitors (PI) are used [141]. When p17 was injected into the mouse hippocampus, it was observed to colocalize with plaques, phosphorylated Tau and fibril-like structures. In the same study, p17 was further demonstrated to be associated with increased Aβ production and impairment of cognitive function in experimental tests [141]. Recently, the regulatory effect of Gag polyprotein on AβPP metabolism has been demonstrated in macrophages and microglia. The Gag enhances Aβ load and associated neurotoxicity by triggering the activity of secretases. AβPP, on the other hand, mediates antiviral actions by sequestering Gag polyprotein in lipid rafts and limiting the release of HIV-1 [142]. To understand the balance between these two mechanisms (envision and restriction), and the impact on toxic Aβ peptide production, further studies are warranted.

The role of Tau protein in HAND pathogenesis is yet to be understood well. However, cognitive abnormalities accompanied by neuronal death and gliosis as a result of Tau hyperphosphorylation have been reported in transgenic mice (10-month-old Gp120 transgenic mice) [112]. Over-activation of glycogen synthase kinase 3β (GSK-3β) is believed to play a key role in such impairment as it is the main enzyme involved in Tau phosphorylation. Similarly, higher expression of cyclin-dependent kinase 5 (Cdk5), another important enzyme involved in Tau phosphorylation, has also been shown in HIV-1 transgenic rats along with raised levels of pTau (p-Thr181, p-Thr231 and p-Ser396), particularly in the hippocampal components [113]. Observations of experimental models therefore demonstrate the linkage between raised pTau and irregular NFTs in HIV^+^ patients with HAND [20,112,120].

## 6. Correlation between BBB, HIV and AD pathogenesis

BBB dysfunction is often associated with the pathogenesis of various neurodegenerative conditions, including HAND [143,144]. In AD, the micro-vessel disruption has been shown to be consistent with disease onset and progression [145–147]. The occurrence of impaired BBB is shown to be associated with Aβ aggregation in several animal models as well as in patients suffering from AD [148–150]. The BBB impairment arising from HIV-1 infection is probably accountable for the transmission of the virions from the vascular compartments. Additionally, it also proved to boost recruitment of immune cells and facilitates CNS infection by many opportunistic microbes [131,143,144]. The interaction between BBB and HIV-1 may occur in the neurovascular unit (NVU) cells by engaging viral proteins. Some studies have shown that by dysregulating gap junctions, HIV-infected astrocytes can damage BBB integrity and impair brain homeostasis [76]. Numerous viral proteins, including Tat, Gp120, Vpr and Nef, have been found to be associated with deregulated molecular and cellular pathways, and impairing the repair mechanisms, leading to BBB dysfunction [5]. The direct regulatory effect of Tat protein on endothelium has also been shown through multiple cellular routes, such as inhibition of the Ras pathways, culminating in reduced tight junction (TJ) protein expression and BBB dysfunction [151–153]. These effects, mainly triggered by toxic Aβ accumulation in the brain, highlight a direct involvement of HIV proteins in Aβ–BBB interaction. Most importantly, Tat also regulates the expression of various Aβ associated receptors and transporters, which are engaged in the bidirectional movement of peptides across BBB. Recently, it has been shown that extracellular Tat induces receptor for advanced glycation endproducts (RAGE) activity and results in the activation of Ras/MAPK signalling cascade and agglomeration of Aβ [7,152]. In addition, it also reduces the clearance of Aβ across the endothelial cells and inhibits the synthesis of low-density lipoprotein receptor-related protein-1 (LRP-1) [152]. Similar to Tat, Gp120 has shown to alter BBB dynamics by regulating protein kinase C (PKC) and JAK/STAT signalling. Gp120 also increases monocyte migration, through which it enhances the number of HIV-infected monocytes that can cross the BBB to enter the CNS [5,154,155]. On the contrary, recombinant Gp120 administration showed injury in CNS micro-vessels that reveal that Gp120 may directly alter the function of endothelial cells in the brain and influence BBB dynamics [156]. These mechanisms ultimately lead to the diminished clearance of Aβ from the interstitial fluid and thus culminate in Aβ deposition, as well as accumulation in the brain. In this context, it is imperative to reasonably speculate and articulate the intriguing role of the BBB in AD and HAND pathogenesis [150].

## 7. Pathological hallmarks of AD: possible role of HIV

### 7.1. Amyloid beta (Aβ)

Atypical Aβ build-up is an important trait of AD reported in HIV-infected individuals [120,123]. Abnormalities associated with Aβ burden are more frequent in the AD brain than HIV, predominantly in the younger HIV-infected individuals. Ageing is considered as a potential risk for Aβ aggregation in HIV-infected individuals, although recent studies advocate that HIV and ageing both can influence Aβ aggregation independently, as well as together [136]. It has been shown that in HIV-infected individuals, the plaques are typically dispersed, and accumulation of Aβ generally occurs in brain somas and extracellular plaques as well as axonal tracks [120,123,157]. However, in AD, the plaques are of neurotic occurrence, predominantly in the extracellular spaces [158]. Some neuropathological findings demonstrate that Aβ aggregates in HIV cases preferentially in the basal ganglia, frontal lobe and hippocampus [123,159]. Though the site of Aβ deposition may show a discrepancy in AD brain, it usually tends to arise primarily in neocortical areas [158]. There are numerous studies that highlight the connection between long-term cART usage and aggregation of Aβ [123,159]. Accumulated Aβ may also exist without cognitive impairments in older adults; however, it is widespread and ubiquitous in the AD brain, and it is not a central feature of normal cognitive ageing [160]. The Aβ accumulation develops gradually with reduced neurotoxicity in similar brain areas with healthy ageing as in AD [161]. Though Aβ is strongly linked with AD, substantial evidence is still limited in context to HAND, where Aβ assists as a driving force.

### 7.2. Hyperphosphorylated Tau (pTau)

Tau is a microtubule-associated protein (MAP) that is accountable for maintaining a normal neuronal network. Hyperphosphorylation of Tau leads to its dissociation from microtubules and the dissociated tau forms paired helical filaments (PHFs) that eventually aggregate and generate NFTs. NFTs consisting of pTau are another characteristic trait of AD, specifically in people suffering from HIV [3,162,163]. The elevated level of Tau has been reported to occur at earlier ages in individuals suffering from HIV than in healthy individuals [20]. Even though pTau contents were found to be irrelevant to the viral levels in the brain, but pTau is often correlated with the activation of microglia [21]. In HIV cases, tau phosphorylation may be initiated by viral proteins as well as pro-inflammatory cytokines that cause amyloidosis and precede the growth of tau tangles [11]. Higher expression of pTau has also been shown to be correlated with ARV treatment [20]. It has been observed that relative to HIV, pTau usually forms in the entorhinal cortex and hippocampus, and later expands to adjacent areas, which represents the phenomenon observed during natural ageing and AD [20,164].

### 7.3. BBB impairment

The BBB is a biochemical barrier that helps in protecting CNS from potentially damaging substances, including neurotoxins and drugs. It also protects the neural tissues from variations in blood composition and neurotoxins [162]. The permeability of the BBB is altered in HIV infection, which permits effusion or leakage of toxic elements, such as infected macrophages from blood to the brain parenchyma. HIV has been reported to influence neuronal endocytosis, which further serves as a key player in impairing the integrity of BBB associated microvascular endothelial cells [165]. Further, upregulation of adhesion molecules and HIV-induced damage of the tight cell junctions facilitate BBB passage [6]. The disrupted BBB has also been correlated with toxic Aβ aggregation in HIV-infected individuals as other abnormalities arise from functional failure to sort out the Aβ peptides [7]. The increased intracellular Aβ agglomeration in microvascular endothelial cells has also been shown during HIV infection in an *in vitro* study [166]. The disrupted BBB, which is linked with AD pathogenesis, serves both as a reason and mediator of cerebral Aβ deposition affecting BBB permeability and Aβ agglomeration involving a common pathophysiological mechanism in AD and HIV cases [7,167].

### 7.4. CSF markers

The phosphorylated Tau and Aβ concentrations in CSF also correspond with their levels in the brain, though for toxic Aβ an opposite correlation exists, indicating a problem that is associated with its Aβ clearance. The higher expression of pTau and reduced Aβ level have been reported in the CSF of individuals suffering from symptomatic HIV, representing the phenomenon observed in AD. However, this finding lacks consistency principally for total Tau and pTau [120,168]. In a study, reduced CSF Aβ, but not accelerated pTau, was observed in an individual suffering with HAND [169]. Conversely, accelerated CSF pTau was also noted in asymptomatic HIV patients as compared to the normal controls [170]. Further, this finding also indicates raised levels of CSF pTau in HIV-infected older people suffering from HAND. In view of this finding, it is seen that similarities exist between HIV^+^ individuals and AD brain with reference to CSF Aβ and Tau, although larger disturbances have been observed consistently during AD in older people, predominantly in comparison with young adults manifesting neuro-asymptomatic HIV.

## 8. Risk factors and pathophysiological mechanisms of AD induced by HIV

### 8.1. Genetic predisposition

The apolipoproteins, in particular *ε*4 allele of apolipoprotein-E (ApoE*ε*4), is known to be one of the major risk factors for AD, which is correlated with elevated Aβ agglomeration, diminished neurocognitive activity, decreased brain volumes and enhanced systemic progression of HIV infection [171–173]. ApoE*ε*4 susceptibility to HIV infection has been shown to be enhanced *in vitro* [173]. The greater expression of ApoE*ε*4 was shown to be correlated with decreased cognition in HIV cases when compared with age-matched seronegative ApoE*ε*4+ individuals, though many studies did not find a meaningful correlation between ApoE*ε*4 and HAND [172,174]. Another isoform, ApoE*μ*4, has been shown to display a more stable association with cognitive functioning in AD than in HIV cases, as evidenced by the fact that carriers with two alleles may have up to 85–90% probability of developing AD by the age of 80. Many risk factors associated with developing AD have also been reported with the ApoE*ε*4 risk alleles [171]. Although HIV may influence neurological structure and function, aggravated by pre-existing genetic factors, and then eventually lead to neurodegeneration or cognitive dysfunction following epigenetic changes [175].

### 8.2. Cerebral metabolism

Emerging evidence shows that HIV infection in individuals causes disturbances in cerebral metabolism, which significantly contributes to the development of brain defects and progression of neurocognitive deficit [6,176,177]. In HIV infection, there is mitochondrial dysfunction followed by oxidative stress via overproduction of reactive oxygen species (ROS), the release of neuroinflammatory markers, neuroimmune dysfunction, susceptibility to drug toxicities and development of HAND [6,177,178]. ROS is considered as the main cause of brain ageing due to oxidative changes as well as cellular damage that affects the aged brain along with impaired insulin signalling [179,180]. Further, glutamate overproduction, enhanced neuroinflammation and Ca^2+^ overload is associated with mitochondrial dysfunction, and all these contribute to the neurotoxicity [181]. Likewise, perturbations in brain mitochondrial activity, oxygen utilization capacity and carbohydrate metabolism have also been implicated in AD [182,183]. Additionally, the occurrence of oxidative stress at an early stage of AD promotes and facilitates the formation of Aβ-plaques and tau tangles [182].

### 8.3. Neuroinflammation

The dispersal of HIV takes place between infected monocytes to uninfected cerebral microglia and astrocytes, where it activates inflammatory immune responses by releasing cytokines, chemokines and ROS. Chronic and sustained neuroinflammation caused by prolonged glial and astrocyte activation has been reported to culminate in neuronal death and exhibit correlation with brain defects associated with HIV infections [6,177,178]. The positron emission tomography (PET) results have also shown functional changes due to regional microglial activation, consistent with autopsy findings that demonstrate frontal cortical aggregation of oxidative damage of macromolecules initiated by ROS in AIDS patients [184,185]. Enhanced glial expression has been observed in asymptomatic neuro cases of HIV with substantial activation of frontal and parietal components among people with HAD. This demonstrates that excessive glial activation and neuroinflammation attribute to cognitive impairment [186]. PET results also indicated that the systemic stimulation of microglia occurs in AD, often in conjunction with cognitive impairment [187]. Aβ aggregation also contributes to astrocyte activation as well as the onset of inflammatory reactions and related immunological responses. In addition to Aβ accumulation, NFTs induced neuronal degeneration also provokes neuroinflammation [167].

### 8.4. Neurotoxicity

An orchestrated reaction of excitotoxicity and apoptosis, which maintains immunological and inflammatory responses to the virus is potentially accountable for HIV-related brain dysfunction [6,177,180]. It has been found that depletion of T-cells and apoptosis are influenced directly by HIV gene expression, whereas indirectly by apoptosis in the uninfected cells. Tat, Gp120 and complementary proteins (such as Fas) are among the substances that have been implicated in HIV-associated neurotoxicity. Tat and Gp120 disrupt the uptake of glutamate by astrocytes, leading to glutamate excitotoxicity and trigger neuroinflammation and apoptosis. Further, they also result in Ca^2+^ accumulation and have neurotoxic effects of a related kind. Moreover, Tat can promote astrocytosis and neuronal death and associate with AβPP to enhance Aβ production [124]. Most importantly, viral structures and regulatory proteins also contribute to cerebral mitochondrial damage and BBB dysfunction following overproduction of ROS that causes oxidative injury [178,188].

Neurotoxicity may also result from numerous ARV drugs used to treat HIV cases, such as nucleoside analogue reverse transcriptase inhibitors. Some ARV drugs that penetrate the BBB and enter the brain efficiently than others possess more potential to cope with HIV-associated brain dysfunction [189]. In recent trials, cART-treated HIV patients exhibited a higher concentration of cerebral Aβ as well as pTau than cART-naive patients [20,123]. There have been contradictory results, but it seems unlikely that cART tends to be the major reason for brain dysfunction in most cases [169,190]. Nevertheless, further studies are required on cART-related neurotoxicity; specifically provided ongoing usage of cART in people of old age suffering from HIV and the probability of emergence of many medications which are under the different stages of clinical development. The inflammation and infection of other organ systems outside of the brain, including liver, gut and vascular systems may also represent indirect neurotoxicity. For instance, HIV causes leaky gut syndrome by damaging and impairing the permeability of the intestinal lining, allowing microbes and toxins to enter the blood and reach systemic circulation, which eventually causes neuroinflammation [191]. Further, in response to HIV, hepatic ceramides were correlated with various components of the metabolic syndrome, apoptosis and neurodegeneration [192].

### 8.5. Vascular and metabolic comorbidities

Numerous comorbidities like chronic substance abuse, often independent of the direct consequences of HIV, lead to HIV transmission, responsiveness and cognitive difficulties [22]. HCV also aggravates HIV-associated neurocognitive damage following similar mechanisms [193,194]. Further, vascular and metabolic conditions such as metabolic syndrome, diabetes mellitus, vascular injury and obesity are in parallel rise with chronically HIV-infected people age, and there are indications that HIV permits them to improve and flourish [195,196]. These conditions can also have an adverse effect on neurocognitive function [197,198]. For instance, impaired glucose metabolism which results in hyperglycaemia and hyperinsulinaemia provokes ROS production, tau hyperphosphorylation, Aβ accumulation and brain microangiopathy, and altogether these contribute towards a reduction in Aβ degradation and clearance [197]. Hence, vascular, neurological dysfunction may be a significant component of HAND caused by HIV, along with the development of vascular comorbidities. However, it is still challenging to identify the specific effect of vascular cognitive dysfunction to HAND. It should also be underlined that vascular risk factors are strongly dominant in aged individuals and there is a strong indication that these risk factors can be correlated with vascular, neurological impairment, even though there are no distinct cerebrovascular events [199]. Further, the epidemiological studies also suggest that these conditions raise the possibility of progression of AD and increase vascular risk in both HIV and AD individuals, and are correlated with higher Aβ burden [198,200–202]. Additionally, the flexible complexity of vascular and metabolic risk factors may essentially represent therapeutic targets in order to prevent or curtail cognitive impairments in HIV-infected individuals.

## 9. Possible mechanisms linking HAND, synaptic degeneration and AD

**Figure 3.**
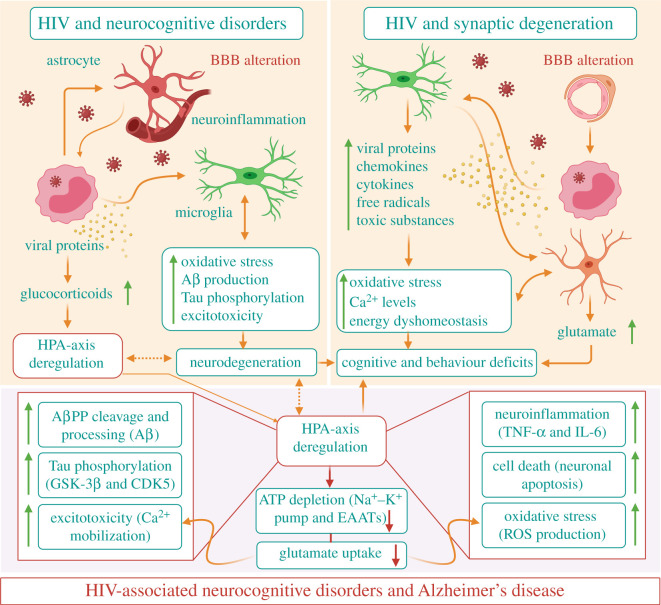
Schematic showing possible linkage between HAND, synaptic degeneration and AD.

As described previously, HIV-1 infection of the CNS initiates from the transmigration of HIV-1-infected peripheral blood monocytic cells/macrophages across the BBB. Subsequently, microglia and astrocytes become infected and reactivated. The immune-activated and HIV-1-infected microglia/macrophages release viral proteins (e.g. gp120, Tat, Nef and Vpr), chemokines (e.g. MCP1, CXCL12), cytokines (e.g. IL-1β, TNF-α, IL-6) and other neurotoxic factors. In addition, infected/reactivated astrocytes can also release neurotoxic substances and pathogenically increase synaptic activity with increased transmitter release and impaired glutamate re-uptake. The released neurotoxins and extracellular glutamate can cause excessive Ca^2+^ influx, perturbations of energy metabolism and ROS production, leading to the disruption of normal neuronal function. Most importantly, the released viral proteins, cytokines, chemokines and free radicals can trigger more glial cells and macrophages. These damaged neurons may mark the abnormal synapses with some kind of ‘eat-me’ signals, which can be recognized and eliminated by microglia and/or astrocytes through phagocytotic pathways such as the MerTK, Megf10 and APOE pathway in astrocytes and the complementary and FKN/CX3CR1 pathways in microglia. Further, all these mechanisms can contribute to AD-like characteristics, including Tau phosphorylation, Aβ production, oxidative stress and excitotoxicity, and also influence neuron integrity and CNS homeostasis. It is also observed that HIV^+^ patients present high glucocorticoid (cortisol) levels, characteristic of a hypothalamic–pituitary–adrenal (HPA) axis deregulation. Glucocorticoids (GC) and their receptors are highly engaged in the etiology of AD. Further, GC and their receptors may modulate/potentiate the development of HAND and potentially AD. The dysregulation of the HPA axis is observed both in HIV^+^ individuals and rodent models. GC overexposure, along with viral proteins or not, is able to induce the enhancement of Tau phosphorylation, Aβ production, oxidative stress, excitotoxicity, neuroinflammation and apoptosis. Through these numerous pathways, HIV-1 causes synaptic deficits and neurodegeneration, thus leading to cognitive impairment and behavioural deficits, and could also explain the establishment of HAND in HIV^+^ patients, and potentially the onset of AD. All these processes lead to neurodegeneration and synaptic deficits/degeneration, and are potentially responsible for cognitive decline observed in HAND patients, all of which could progressively favour the development of AD (figure 3) [203,204].

## 10. Therapeutics strategies to combat HIV-mediated neuronal damage

**Figure 4.**
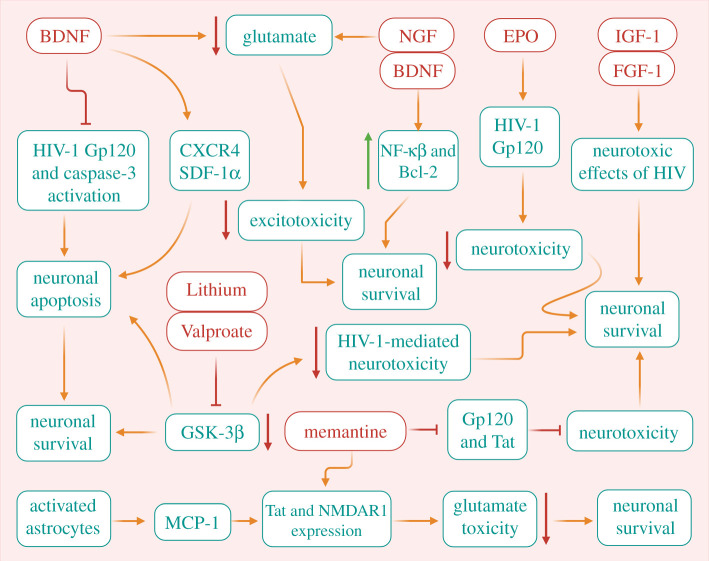
Proposed protective role of neurotrophins, growth factors and drugs in combating HIV-induced neuronal damage. A neurotrophic agent such as BDNF confers neuroprotection via inhibiting caspase-3 activation and HIV-1 gp120 mediated neuronal apoptosis. BDNF can also curtail the levels of CXCR4 and prevent neuronal apoptosis by blocking the neurotoxic effects of SDF-1α. NGF and BDNF mediated activation of NF-κβ and upregulation of Bcl-2 may act as another way to promote neuronal survival in HIV-1 associated neurodegeneration. In addition, NGF signalling components can also prevent glutamate-induced neurotoxicity induced by ischaemic injury. Similarly, a neurotrophin like EPO has potential to protect cortical neurons against apoptosis by targeting HIV-1 gp120. Since impaired IGF-I and FGF responses were recorded during the course of HIV infection in several studies, therefore higher expression of these factors can also rescue the CNS from the neurotoxic effects of HIV. The altered expression of FGF-I and GSK-3β in susceptible neurons are now regarded as crucial during HAD pathogenesis. Further, drugs like memantine can be used to prevent neurotoxicity induced by Tat and gp120 viral proteins. Similarly, using inhibitors/drugs like valproate and lithium for GSK-3β could have therapeutic importance in HAD patients, since higher expression of GSK-3β induces apoptosis and it has been found to be associated with HIV-1 protein-mediated neurotoxicity. Finally, activated astrocytes-induced MCP-1 production positively influences neuroprotection through caspase-1 blockade. On the contrary, MCP-1 associated inflammatory reaction contributes to HIV-1- associated neurological illness. MCP-1 can protect human mixed cultures of neurons and astrocytes from Tat or NMDA-induced apoptosis by downregulating the extracellular glutamate expression, and in neurons by modulating Tat and NMDAR1 expression. These strategies together can be helpful in preventing HIV-induced neuronal damage.

In the above sections, we comprehensively discussed various underlying interconnected mechanisms between HIV, neuroinflammation, HAND and AD. Understanding the underlying mechanisms will help explore various possible therapeutic strategies and agents, which may be able to combat these complications. Unfortunately, there are no medications identified so far, and very few studies are available on therapeutic aspects. Neuroprotective therapies are designed with a targeted approach to ameliorate damage and improve survival as well as the function of neurons. The mechanisms associated with neuroprotection are classically aimed to diminish the extent of neuronal damage in HIV-1-induced neuronal dysfunction. It can be considered that agents that regulate inflammatory and/or cell death pathways and favourably modulate neurotransmitter function may provide opportunities for pharmacological manipulation during HIV-1 brain infections, although previous studies which focused on anti-inflammatory mechanisms have not demonstrated promising results in attenuating endogenous inflammation and considerable neuroprotection. As a result, a number of studies have recently been conducted to reduce neurotoxicity by blocking or modulating the actions of viral proteins, augmenting the protective action of neurotrophins and growth factors, or curtailing neuroinflammation triggered by HIV-1-infected microglia and macrophages (figure 4). For instance, the neuroprotective role of brain-derived neurotrophic factor (BDNF) has recently been observed in HIV-1-mediated neurotoxicity. It appears a potent neurotrophic agent for HIV-1 associated neuronal injury, which confers neuroprotection via inhibiting caspase-3 activation and HIV-1 Gp120 mediated neuronal apoptosis [205]. Moreover, BDNF is also found to reduce the levels of CXC chemokine receptor- 4 (CXCR4) and inhibit neuronal apoptosis by blocking the neurotoxic effects of SDF-1α, a ligand for CXCR4. The SDF-1-mediated apoptosis is quantitatively akin to that provoked by Gp120. CXCR4 activation can contribute to the cell death of a different kind of neuronal population. Consequently, BDNF-mediated neuroprotection occurs by reducing CXCR4 level that ultimately leads to the reduced activation of this receptor during HIV-1 neuropathogenesis [205]. Recently, activation of nuclear factor kappa beta (NF-κβ) mediating nerve growth factor (NGF) and BDNF and rise in Bcl-2 expression has also been reported to promote neuronal survival in HIV-1 associated neurodegeneration [206,207]. Additionally, BDNF has also been reported to prevent glutamate-induced excitotoxicity through modulation of NMDA receptors in HIV-1 patients [208]. Similarly, erythropoetin (Epo), a neurotrophin, can also confer neuroprotection against HIV [209]. A higher dose of Epo for a long duration showed better neuroprotective effect against HIV-1 transmission from mother to infant [210]. It can also protect cortical neurons against apoptosis by targeting HIV-1 Gp120 [211]. These observations suggest that Epo can be considered as a potential therapeutic agent for the treatment of HAD [212]. Recently, the promising role of recombinant human NGF (rhNGF) has shown to improve the symptoms associated with both HIV-related neuropathy and diabetic polyneuropathy. Substantial evidence demonstrates that NGF signalling may also prevent glutamate-induced neurotoxicity caused by ischemic injury. However, in HIV-1-induced neuronal damage, especially in the peripheral nervous system, NGF may have significant therapeutic effects [213–215]. Activation of the insulin-like growth factor I (IGF-I) system is another potential approach to treat HAD, as it exhibited neuroprotective action against neurotoxins [216–218]. Activating IGF-I-stimulated signalling components may offer a potential therapeutic approach to protect susceptible neurons in HAD patients. Earlier, impaired IGF-I responses were reported during the course of HIV infection [216–218]. In HIV-infected patients, reduced levels of serum IGF-I have been observed particularly in children failure to thrive and individuals displaying wasting syndrome [216]. Reduction in the levels of IGF-I in CNS may aggravate neuronal apoptosis in the course of HIV infection [218]. Thus, it can be reasonably argued that activation of the IGF-I system or increased utilization of IGF-I-activated pathways may signify a promising treatment approach to rescue neurons susceptible or vulnerable to injury in HAD patients. Similarly, higher expression of fibroblast growth factor I FGF-I can also rescue the CNS from the neurotoxic effects of HIV. Altered expression of FGF-I and GSK-3β in susceptible neurons can be considered crucially important for the pathogenesis of HAD and emergence of therapeutic strategies [219,220].

Furthermore, the Tat and Gp120 mediated neurotoxicity can be fully blocked by memantine, an NMDA antagonist used well in the treatment of dementia [221,222]. It also ameliorates hippocampal synaptic transmission in the SCID mouse model of HIV-1-associated neurologic diseases [223]. Recently, the use of inhibitors of GSK-3β in the brain suggested that regulation of GSK-3β activity in neurons may be vital for neuroprotection. Higher expressions of GSK-3β induced apoptosis and showed association with HIV-1 protein-mediated neurotoxicity [224,225]. As a consequence, pharmacological agents like valproate and lithium identified to inhibit GSK- 3β activity could be valuable for therapeutic benefits in HAD patients.

The neuroprotective role of monocyte chemoattractant protein 1 (MCP-1) has recently been observed in HIV and HAD patients [226,227]. Activated astrocytes-induced MCP-1 production positively influences neuroprotection through the caspase-1 blockade. On the contrary, MCP-1 associated inflammatory reactions contribute to HIV-1-associated neurological ailments [226,227]. MCP-1 can protect mixed cultures of neurons and astrocytes from Tat or NMDA-induced apoptosis by downregulating the extracellular glutamate expression, along with modulating Tat and NMDAR1 expression [228]. In the case of HAD, MCP-1 may exert a protective as well as a degenerative role as it is coupled with monocyte recruitment and inflammation into the CNS [229]. The intricate balance between neuroinflammation and neuroprotection could be vital in triggering the initial as well as the ongoing response of the CNS to injury. Taken together, potential approaches to amplify the biologic effects of these factors or intensify their expression may support an advantageous role against this type of neurodegeneration.

## 11. Antiretroviral drugs: potential therapeutic agent for the treatment of HIV-induced neuronal damage

More recently, drugs used in highly active antiretroviral therapy (HAART) have shown improvement in cognitive functions, including all cognitive paradigms. The cognitive improvement is also correlated with an increase in CD4 count with a concomitant reduction in viral load [230]. The ability of ARV drugs to penetrate CNS supports the basis of its therapeutic success, as is evident in various reports. In order to reduce viral load, it is important that the drug should achieve a high concentration in the CSF following its ability to cross the BBB. Letendre *et al.* [231] examined the CNS penetrability of ARV drugs and ranked the ARV drugs for penetration based on scores assigned as 0 (low), 0.5 (intermediate) or 1 (high). This ranking system was based on drug concentrations in CSF, effectiveness in CNS and chemical properties in the clinical studies. The calculation for CNS penetration effectiveness (CPE) rank was determined by summing the individual penetration ranks for each ARV in the regime. For instance, combinations of efavirenz, zidovudine and lamivudine scored high for CPE [232]. Drugs like abacavir displayed low CPE score and rank; this was correlated well with higher viral load in the CSF [232]. Moreover, a small study involving 37 individuals demonstrated greater cognitive improvement with higher drug penetrability [233]. Similarly, another study looked at both HIV patients with cognitive impairment and patients with cognitive impairment without HIV, and it showed a worsening of cognitive. The ARV drugs with high penetrability can be neurotoxic too; thus, it is advised to suspect ARV drug neurotoxicity when cognitive improvement is not observed or detected with ARV treatment [234].

In the last few decades, appreciable progress has been made in the area of ARV therapy related to improved neurological clinical outcomes for HIV-1 patients. An immediate first-line treatment regimen for all new diagnosed HIV-1 infected patients is recommended by international guidelines for reducing the neurological complications associated with HIV-1infected patients [235,236]. Current ARV therapy is highly efficient in controlling HIV-1; still, viral replication can be found in the CSF among some patients. It has been found that ARVs reach different areas of CSF with significant variability due to the different expression profiles of cellular drug transporters and the concentrations of few ARVs do not the exceed inhibitory concentration for wild-type HIV replication in CSF [237,238] (table 4). The main limitation to achieve the HIV-1 eradication from the brain is the suboptimal concentrations of ARV within this site. Factors like molecular weight, blood protein binding and lipophilicity influence the concentration of drug in the brain tissue [231,241–243]. For instance, while entry and integrase inhibitors are able to reach the CNS, the nucleoside/nucleotide reverse transcriptase inhibitors and non-nucleoside reverse transcriptase inhibitors can only partially cross the BBB. Conversely, the majority of PIs are characterized by a medium/low permeability to the BBB [5,239,244,245]. Furthermore, some cellular transporters like P-gp, MRP4 and MRP5 have the ability to reduce the intracellular concentration of ARV drugs which ultimately favours both the emergence of drug-resistant viruses and their productive infections to other cells [46,56,240,246].

**Table 4.** Class, name and CNS penetration of the antiretroviral drugs [239,240].

class of drug	name of the drug	CNS penetration
protease inhibitor	tipranavir	low
	fosamprenavir	medium
	atazanavir	medium
	saquinavir	low
	nelfinavir	low
	lopinavir	medium
	ritonavir	low
	darunavir	medium
	indinavir	medium
	amprenavir	medium
nucleoside reverse transcriptase inhibitor	tenofovir disoproxil fumarate	low
	abacavir	medium
	didanosine	medium
	emtricitabine	medium
	stavudine	medium
	lamivudine	medium
	zidovudine	high
entry/fusion inhibitors	maraviroc	high
	enfuvirtide	low
non-nucleoside reverse transcriptase inhibitor	etravirine	low
	delavirdine	high
	nevirapine	high
	efavirenz	medium
integrase strand transfer inhibitor	raltegravir	medium
	elvitegravir	medium

New strategies like the usage of a hypertonic solution of urea or mannitol [48,49] are currently used to increase the concentrations of ARV within site. This deed can be achieved by inhibiting the drug efflux transport, while nanoparticles and cell-mediated nanoART may confer other key advantages, such as improved blood half-life and bioavailability, precise delivery and higher aqueous stability [231]. Different types of nanoparticles that have been identified for improving the concentration of ARV are listed below:

1. Lipid nanoparticles have the ability to easily cross the BBB [247,248].
2. Polymeric nanoparticles are able to exploit the interaction with low-density lipoproteins receptors on the surface of endothelial cells [239,249].
3. Inorganic nanoparticles such as small size silica with the addition of polyethylene glycol (PEG) [250].
4. Gold nanoparticles conjugated with cell-penetrating peptides [251].

It has been recently reported that poly(dl-lactide-co-glycolide) nanoparticles and other nanoparticles increase the peak concentrations of lopinavir, ritonavir and efavirenz (these drugs are characterized by a low penetration into CNS) [239,252]. Recently, a CPE that depends on pharmacokinetics' features of various ARV drugs was proposed to estimate the efficacy of ARV treatment in the CSF [238]. However, some contradictory results of this CPE on clinical outcomes in HIV-1 infected patients have been reported in some of the studies [169,232,234]. These observations reflect that further studies are required to prescribe ARV therapy and that the regimens characterized by high CPE scores must be carefully chosen. It has been demonstrated that in the presence of high CPE, there is an acceleration of neurological disorders [253,254]. For instance, PIs are shown to induce oxidative stress in neuronal cells, while the NNRTI efavirenz caused toxicity in the cortical neuronal cultures of fetal rats [253–255]. Still *in vivo* studies are needed to confirm the neurotoxicity profiles of these drugs for potential applications.

Further, various reports highlighted the use of psychiatric medication for mood disorders like depression. Many subtypes of antidepressants, including tricyclic antidepressants, serotonin–norepinephrine re-uptake inhibitors and selective serotonin re-uptake inhibitors, have been found useful in providing moderate symptomatic relief [256,257]. Psychostimulants may also be useful for apathy and fatigue [258]. Psychotic and manic symptoms are less reported in the case of HIV^+^ individuals, though a small-scale study with psychosis demonstrated a higher occurrence of extrapyramidal symptoms [259]. Numerous drugs such as mood stabilizers (like lithium) may have concurrent neurotoxic effects, and carbamazepine may stimulate the same CYP enzyme system which participates in the metabolism of ARV drugs, and therefore may cause drug–drug interactions [260,261]. However, on a pharmacological basis, many agents including memantine, nimodipine, selegiline, pentoxifylline and peptide T can be considered neuroprotective, although among these numerous agents, only selegiline appears to exhibit potential benefits [262].

## 12. Conclusion

Based on the available literature, it can be concluded that HIV-associated synaptic loss and aetiology of AD and HAND is an interconnected and orchestrated consequence of numerous neuropathogenic processes triggered by HIV-1. Interactions between HIV-1 and the host cells are believed to play a vital role in the pathogenesis of these abnormalities. Several viral proteins (Tat, Gp120, Nef and Vpr), which are released from infected cells in the nervous system, may impart induction of synaptic injury and pathogenesis of AD. In addition, these proteins are likely to act in conjunction and cause synaptotoxicity when released from infected cells in the CNS. Further, AD-associated numerous factors such as BBB regulators, members of the stress-related pathways as well as the amyloid and Tau pathways appear to augment amyloid plaques deposition or NFT accumulation following HIV neuroinfections. Additionally, the HPA axis dysregulation also showed that when associated with HIV infection, it is conducive of generating an environment where BBB disruption, neuroinflammation, oxidative stress, excitotoxicity and Aβ burden are exacerbated. This, combined with other factors (environmental/genetic), may provide a new insight for understanding the pathogenesis, diagnosis and therapeutics of brain disorders including AD and HAND. The scenario of replication-independent production of HIV-1 protein is apparently counterintuitive, and the underlying molecular mechanism is yet largely remained unexplored. It is therefore imperative to explore more in this field. Considering the need for therapeutics against HIV neuroinfection, unfortunately, still, there is an urgent need for evidence-based medications to be identified that would be able to combat these complications. Many studies have recently shown a reduction in neurotoxicity via modulating the actions of viral proteins, augmenting the protective action of neurotrophins and growth factors, or curtailing neuroinflammation triggered by HIV-1-infected microglia and macrophages. The mechanisms associated with neuroprotection are classically aimed to diminish the extent of neuronal damage in HIV-1-induced synaptic dysfunction. Agents that regulate inflammatory and/or cell death pathways and favourably modulate neurotransmitter function may provide opportunities for pharmacological manipulation during HIV-1 brain infections. Altogether, in the near future, it could be of paramount significance to explore the molecular mechanisms of HIV neuroinfection and develop therapeutic strategies.

## Data accessibility

This article has no additional data.
